# Relationship between ankle mobility and vertical jump performance across playing positions in professional female volleyball players

**DOI:** 10.3389/fspor.2026.1879769

**Published:** 2026-09-15

**Authors:** Damjana V. Cabarkapa, Dimitrije Cabarkapa, Vladimir Bankovic, Nikola Majstorovic, Thayne A. Munce

**Affiliations:** 1 D2 Lab, Novi Sad, Serbia; 2 Faculty of Physical Culture and Sports Management, Singidunum University, Belgrade, Serbia; 3 Faculty of Sport and Physical Education, University of Belgrade, Belgrade, Serbia; 4 Department of Health, Sport and Exercise Science, Jayhawk Athletic Performance Laboratory—Wu Tsai Human performance Alliance, University of Kansas, Lawrence, KS, United States

**Keywords:** asymmetry, female athletes, force plate, neuromuscular performance, range of motion

## Abstract

The purpose of this study was to examine the associations between ankle range of motion (ROM), inter-limb ROM asymmetry (ASYM), and countermovement vertical jump (CMJ) force-time metrics across five playing positions in professional female volleyball players (*n* = 124). Following a standardized warm-up protocol, ankle ROM was assessed bilaterally during a weight-bearing front lunge using a digital goniometer. Then, each athlete performed three CMJs with no arm swing while standing on a uni-axial force plate system. Pearson product-moment correlation coefficients were used to measure the strength of linear regressions (*p* < 0.05). When analyzed across all playing positions, ankle ROM demonstrated moderate inverse associations with both eccentric (*r* = −0.297) and concentric peak force (*r* = −0.362). However, the direction and magnitude of the associations tended to differ by playing position. Setters revealed inverse relationship between ROM and eccentric (*r* = −0.442) and concentric peak force (*r* = −0.452), concentric peak power (*r* = −0.497), and jump height (*r* = −0.390). Opposite hitters showed inverse associations between ROM and concentric peak velocity (*r* = −0.631), peak force (*r* = −0.466), peak power (*r* = −0.559), and jump height (*r* = −0.630). Liberos displayed longer eccentric (*r* = 0.390) and concentric phase durations (*r* = 0.469) with greater ankle ROM, alongside lower eccentric (*r* = −0.640) and concentric peak force (*r* = −0.697). No significant associations were observed in outside hitters or middle blockers. Also, ASYM showed no significant relationships with any CMJ force-time metric of interest, cumulatively and within each playing position. Overall, the findings indicate that associations between ankle ROM and CMJ performance varied across playing positions, suggesting that ankle mobility should be interpreted within the context of positional demands rather than as a universal performance determinant.

## 1 Introduction

Volleyball is a high-intensity intermittent team sport characterized by repeated explosive actions such as vertical jumping, rapid accelerations and decelerations, blocking, spiking, and frequent change-of-direction movements (1–4). Among these physical demands, vertical jumping represents one of the most critical performance parameters in volleyball, as it directly influences attack reach height, block effectiveness, and overall tactical efficiency (5–8). To date, match-play analyses have demonstrated that players may perform dozens of jumps per set, with position-specific differences in jump frequency and intensity, reflecting distinct tactical responsibilities (7, 9, 10). Given these substantial physical and physiological demands, monitoring lower-body neuromuscular performance has become an integral component of athlete management process in elite volleyball. Commonly utilized assessments include countermovement vertical jump (CMJ), squat jump, drop jump, and various types of sprint-related tests (e.g., 10 m or 20 m). Among these, the CMJ is one of the most frequently implemented assessment methods due to its ecological validity, practicality, and sensitivity to both training-induced adaptations and fatigue-related changes in performance (7, 11, 12).

Among the biomechanical factors that potentially influence vertical jump performance in volleyball (e.g., tendon stiffness, lower-limb muscle strength, force-time characteristics), ankle joint mobility, particularly dorsiflexion range of motion (ROM), has received significant attention. From a mechanical standpoint, restricted ankle mobility may alter sagittal-plane kinematics, shift load distribution toward proximal joints (e.g., knee and hip), and modify force-time characteristics during both eccentric and concentric phases of the stretch-shortening cycle (13, 14). Conversely, excessive mobility could theoretically reduce joint stiffness and impair efficient force transmission, particularly in tasks requiring rapid force development (15, 16). Previous research conducted in jumping-dominant sports has reported associations between ankle dorsiflexion and vertical jump performance, with some studies demonstrating positive relationships between ROM and vertical jump height, whereas others have reported trivial or even inverse associations (17–20). In volleyball populations specifically, previous research reports have largely been focused on studying youth or amateur cohorts of athletes, and methodological heterogeneity, including differences in ROM assessment techniques and jump outcome measures, has contributed to inconsistent findings (18, 19).

Given that vertical jump performance is most commonly assessed using CMJ, examining the relationship between ankle ROM and CMJ-derived mechanical characteristics is particularly relevant in volleyball populations. The CMJ has been recognized as a valid and reliable indicator of lower-body explosive performance and neuromuscular function in volleyball players (11, 12, 21). Beyond simple measures of jump height, force-plate-based CMJ assessment enables comprehensive evaluation of eccentric (i.e., braking) and concentric (i.e., propulsive) phase durations, peak and mean force and power outputs, contraction time, countermovement depth, and reactive strength index (RSI)-modified (21–23). Combined, these force-time characteristics provide valuable insight into movement strategy and the mechanical determinants of jumping performance. Importantly, volleyball is characterized by distinct positional roles (e.g., setter, opposite hitter, middle-blocker), each associated with unique movement and neuromuscular performance demands (2, 3, 25, 26). For instance, setters frequently perform submaximal rhythm-controlled jumps to optimize tactical ball distribution, whereas liberos predominantly operate in low defensive positions requiring rapid postural adjustments. In contrast, opposite hitters and middle blockers repeatedly execute maximal or near-maximal explosive jumps under strict temporal constraints (2, 3, 25, 26). Thus, given these position-specific mechanical and neuromuscular requirements, the association between ankle ROM and CMJ performance may vary across playing roles and could be moderated by tactical demands and anthropometric profile.

Despite the theoretical importance of ankle ROM in modulating jump mechanics, limited research has examined its relationship with detailed CMJ-derived force-time characteristics in professional female volleyball players. Furthermore, it remains unclear whether these associations differ across playing positions within elite cohorts, where tactical demands and neuromuscular requirements vary substantially. Clarifying these relationships may help determine whether ankle mobility functions as a performance-enhancing attribute, a neutral characteristic once adequate levels are achieved, or a context-dependent variable whose influence is moderated by positional role and mechanical strategy. Therefore, the purpose of the present investigation was to examine the association between ankle ROM and CMJ-derived force-time characteristics across five distinct playing positions in professional female volleyball players.

## 2 Materials and methods

### 2.1 Participants

One hundred and twenty-four professional female volleyball players volunteered to participate in the present study, including *n* = 19 setters, *n* = 20 opposite hitters, *n* = 22 liberos, *n* = 34 outside hitters, and *n* = 29 middle blockers. Anthropometric characteristics across each of the five playing positions are presented in Table 1. All athletes were part of their respective professional volleyball teams competing in the top-tier European league (e.g., SuperLeague), had no history of previous ankle injuries, and were free from any current musculoskeletal injuries that could limit or impair testing procedures. The testing procedures were reviewed and approved by the University's Institutional Review Board, and written informed consent was obtained from all participants prior to data collection.

**Table 1:** Anthropometric characteristics of participants, across each playing position.

Playing position	Age [years]	Body mass [kg]	Height [cm]
Setter	22.1 ± 4.5	71.7 ± 9.8	179.2 ± 3.7
Opposite hitter	23.5 ± 3.7	81.1 ± 6.9	187.6 ± 3.1
Libero	23.8 ± 3.9	62.3 ± 5.8	170.0 ± 6.3
Outside hitter	24.4 ± 4.2	72.4 ± 6.4	184.0 ± 2.9
Middle blocker	22.0 ± 5.4	73.9 ± 6.9	188.7 ± 3.3

### 2.2 Procedures

Following the completion of a standardized dynamic warm-up protocol, composed of dynamic stretching exercises (e.g., A-skip, butt-kicks, side-to-side lunge, high-knees) and low-intensity volleyball-specific drills (10–15 min), athletes completed an ankle ROM test administered by a digital goniometer (K-Move, Kinvent, Montpellier, France). Athletes adopted a lunge stance facing a wall, with the tested foot positioned flat on the floor and the contralateral leg placed behind for balance (i.e., split stance) (26, 27). The device was attached to the dorsal surface of the foot and the anterior aspect of the tibia and calibrated from the upright starting position. Athletes were instructed to advance the knee slowly toward the wall while maintaining heel contact until reaching their maximum pain-free dorsiflexion. The joint ROM was automatically recorded by the goniometer, and each athlete performed two trials with each limb. The average value across both limbs was calculated and used for performance analysis purposes as the representative measure of ankle ROM. Additionally, inter-limb asymmetry (ASYM) was quantified as the absolute difference in ROM between the left and right ankles.

After completion of ROM testing procedures, all athletes completed three CMJs while standing on a uni-axial force plate system sampling at 1,000 Hz (VALD Perfrmance, ForceDecks, Brisbane, Australia). The athletes were instructed to keep their hands on the hips during the entire movement (i.e., no arm swing). A strong verbal encouragement was provided to ensure that athletes were giving maximal effort and were focusing on pushing the ground as forcefully as possible (28). Each jump trial was separated by a 10–15 s rest period to minimize the impact of fatigue, and the mean value across three jumps was used for performance analysis purposes. The system was zeroed between each participant, and the testing procedures were completed 24 h following the last training session of moderate intensity (i.e., rating of perceived exertion 4–6). All testing procedures were completed within the same competitive season span (i.e., pre-season), including athletes competing within the same professional league.

Based on previously published research reports (29–33), twelve force-time metrics of interest were selected for neuromuscular performance assessment, including outcome metrics (e.g., jump height) and strategy metrics (e.g., countermovement depth). As valid and reliable performance parameters, these variables included eccentric and concentric duration and peak velocity, eccentric and concentric peak and mean force and power, contraction time, jump height (i.e., impulse-momentum calculation, reactive strength index RSI-modified (i.e., jump height divided by contraction time), and countermovement depth.

### 2.3 Statistical analysis

Descriptive statistics, means and standard deviations (x¯ ± SD), were calculated for each dependent variable examined in this study. Shapiro–Wilk's test was used to examine the assumption of normality. Pearson product-moment correlation coefficients were used to assess the strength and direction of linear associations between the force-time metrics (e.g., concentric peak force, eccentric peak velocity, concentric duration, vertical jump height) and ROM and ASYM, separately for each variable. The magnitude correlation thresholds were interpreted as follows: *r* = 0.00–0.10 trivial; *r* = 0.10–0.30 small; *r* = 0.30–0.50 moderate; *r* = 0.50–0.70 large; *r* = 0.70–0.90 very large; *r* = 0.91–1.00 perfect (34, 35). Statistical significance was set *a priori* to *p* ≤ 0.05. All statistical analyses were completed with SPSS software (Version 26.0; IBM Corp., Armonk, NY).

## 3 Results

Descriptive statistics, means and standard deviations (x¯ ± SD), for each dependent variable examined in this study are presented in Table 2. When data were pooled across all playing positions, ankle ROM demonstrated statistically significant moderate associations with CMJ force-time characteristics. Specifically, greater ankle ROM was associated with lower eccentric and concentric peak force production. In contrast, ASYM measures were not significantly associated with any force-time metrics, with all observed relationships classified as trivial in magnitude (Table 3).

**Table 2:** Descriptive data for each dependent variable across five distinct playing positions.

Variable [unit]	Overall	Setter	Opposite hitter
ROM [deg]	43.1 ± 6.0	43.8 ± 5.6	40.9 ± 8.1
ASYM [deg]	4.0 ± 2.9	3.0 ± 2.3	4.6 ± 4.0
ECC duration [s]	0.52 ± 0.09	0.51 ± 0.07	0.50 ± 0.08
ECC peak velocity [m/s]	1.19 ± 0.26	1.14 ± 0.26	1.26 ± 0.18
ECC peak force [N]	1,704.1 ± 243.6	1,596.6 ± 277.9	1,740.6 ± 287.7
ECC peak power [W]	1,397.4 ± 446.9	1,217.0 ± 401.7	1,470.8 ± 369.7
CON duration [s]	0.278 ± 0.04	0.27 ± 0.04	0.29 ± 0.03
CON peak velocity [m/s]	2.61 ± 0.17	2.60 ± 0.16	2.64 ± 0.17
CON peak force [N]	1,727.3 ± 226.9	1,616.9 ± 265.7	1,774.2 ± 262.5
CON peak power [W]	3,430.1 ± 482.7	3,207.3 ± 534.2	3,661.2 ± 524.5
Contraction time [s]	0.79 ± 0.11	0.78 ± 0.09	0.79 ± 0.10
Jump height [cm]	32.0 ± 4.6	31.9 ± 4.4	32.6 ± 4.8
RSI-modified [ratio]	0.47 ± 0.09	0.46 ± 0.10	0.48 ± 0.09
CM depth [cm]	29.0 ± 5.7	28.6 ± 6.6	30.5 ± 4.3

	Libero	Outside hitter	Middle blocker
ROM [deg]	43.5 ± 6.5	43.5 ± 4.3	43.2 ± 6.1
ASY [deg]	4.16 ± 2.37	3.80 ± 2.28	4.54 ± 3.59
ECC duration [s]	0.49 ± 0.08	0.55 ± 0.12	0.52 ± 0.07
ECC peak velocity [m/s]	1.19 ± 0.24	1.15 ± 0.30	1.20 ± 0.27
ECC peak force [N]	1,672.3 ± 239.7	1,725.4 ± 232.1	1,746.4 ± 191.9
ECC peak power [W]	1,361.1 ± 405.8	1,439.8 ± 559.5	1,438.4 ± 381.1
CON duration [s]	0.27 ± 0.04	0.28 ± 0.04	0.28 ± 0.04
CON peak velocity [m/s]	2.58 ± 0.15	2.62 ± 0.19	2.61 ± 0.16
CON peak force [N]	1,688.4 ± 231.2	1,744.1 ± 181.9	1,775.2 ± 207.7
CON peak power [W]	3,171.9 ± 334.5	3,481.8 ± 422.1	3,546.4 ± 461.1
Contraction time [s]	0.76 ± 0.10	0.83 ± 0.15	0.80 ± 0.08
Jump height [cm]	31.4 ± 4.1	32.4 ± 5.1	31.7 ± 4.41
RSI-modified [ratio]	0.47 ± 0.07	0.47 ± 0.10	0.46 ± 0.09
CM depth [cm]	28.3 ± 5.3	28.8 ± 5.5	29.1 ± 6.46

CM, countermovement; ECC, eccentric; CON, concentric; ROM, range of motion; ASYM, asymmetry.

**Table 3:** Relationship between countermovement vertical jump force-time metrics within eccentric and concentric phases of the jumping motion and ankle mobility and between-limb asymmetries across all athletes combined.

Variable [unit]	ROM	ASYM
ECC duration [s]	0.055 [0.536]	−0.056 [0.535]
ECC peak velocity [m/s]	−0.003 [0.975]	−0.079 [0.380]
ECC peak force [N]	**−0.297** **[0.001]**	−0.032 [0.719]
ECC peak power [W]	−0.047 [0.604]	−0.015 [0.864]
CON duration [s]	0.079 [0.375]	0.030 [0.736]
CON peak velocity [m/s]	0.174 [0.055]	−0.031 [0.731]
CON peak force [N]	**−0.362** **[0.001]**	−0.064 [0.474]
CON peak power [W]	−0.139 [0.119]	−0.109 [0.223]
Contraction time [s]	0.065 [0.496]	−0.025 [0.779]
Jump height [cm]	0.194 [0.058]	−0.016 [0.857]
RSI-modified [ratio]	0.071 [0.421]	−0.013 [0.887]
CMJ depth [cm]	−0.034 [0.702]	−0.081 [0.367]

Bolded values represent statistically significant relationships (*p* < 0.05).

CM, countermovement; ECC, eccentric; CON, concentric; ROM, range of motion; ASYM, asymmetry.

Following position-specific analyses, setters exhibited a similar pattern, whereby greater ankle ROM was associated with lower eccentric peak force, concentric peak force and power, as well as reduced jump height (i.e., moderate association). Specifically, higher joint mobility values corresponded to diminished force-producing capabilities during both the eccentric and concentric phases of the CMJ. Conversely, between-ankle ASYM measures demonstrated no significant associations with any vertical jump force-time metrics (Table 4).

**Table 4:** Relationship between countermovement vertical jump force-time metrics within eccentric and concentric phases of the jumping motion and ankle mobility and between-limb asymmetries in setters.

Variable [unit]	ROM	ASYM
ECC duration [s]	−0.206 [0.330]	0.149 [0.543]
ECC peak velocity [m/s]	−0.269 [0.266]	0.026 [0.916]
ECC peak force [N]	**−0.442** **[0.046]**	0.202 [0.409]
ECC peak power [W]	0.219 [0.367]	−0.046 [0.853]
CON duration [s]	0.243 [0.315]	−0.074 [0.764]
CON peak velocity [m/s]	−0.392 [0.097]	0.194 [0.427]
CON peak force [N]	**−0.452** **[0.042]**	0.176 [0.472]
CON peak power [W]	**−0.497** **[0.024]**	0.111 [0.652]
Contraction time [s]	−0.077 [0.753]	0.075 [0.759]
Jump height [cm]	**−0.390** **[0.047]**	0.234 [0.334]
RSI-modified [ratio]	−0.148 [0.554]	−0.010 [0.967]
CMJ depth [cm]	−0.140 [0.657]	−0.016 [0.950]

Bolded values represent statistically significant relationships (*p* < 0.05).

CM, countermovement; ECC, eccentric; CON, concentric; ROM, range of motion; ASYM, asymmetry.

Within opposite hitters, a statistically significant moderate inverse association was observed between concentric peak force and ankle ROM. Also, even stronger associations were identified between concentric peak velocity and power and ankle ROM, with greater values being associated with lower ankle mobility. In addition, jump height followed a similar pattern, demonstrating a strong negative association with ankle ROM, whereas the RSI-modified exhibited the same direction of association but of moderate magnitude. In contrast, although between-ankle ASYM measures ranged from small to moderate in magnitude, none reached the level of statistical significance (Table 5).

**Table 5:** Relationship between countermovement vertical jump force-time metrics within eccentric and concentric phases of the jumping motion and ankle mobility and between-limb asymmetries in opposite hitters.

Variable [unit]	ROM	ASYM
ECC duration [s]	0.311 [0.183]	0.211 [0.371]
ECC peak velocity [m/s]	−0.002 [0.992]	−0.067 [0.779]
ECC peak force [N]	−0.334 [0.150]	−0.319 [0.171]
ECC peak power [W]	−0.159 [0.503]	−0.302 [0.196]
CON duration [s]	0.045 [0.851]	0.389 [0.090]
CON peak velocity [m/s]	**−0.631** **[0.003]**	−0.052 [0.828]
CON peak force [N]	**−0.466** **[0.047]**	−0.358 [0.121]
CON peak power [W]	**−0.559** **[0.010]**	−0.157 [0.509]
Contraction time [s]	0.258 [0.272]	0.288 [0.218]
Jump height [cm]	**−0.630** **[0.030]**	−0.033 [0.895]
RSI-modified [ratio]	**−0.458** **[0.042]**	−0.258 [0.274]
CMJ depth [cm]	−0.232 [0.318]	−0.412 [0.071]

Bolded values represent statistically significant relationships (*p* < 0.05).

CM, countermovement; ECC, eccentric; CON, concentric; ROM, range of motion; ASYM, asymmetry.

For liberos, slightly different trends were observed. Eccentric and concentric phase durations demonstrated statistically significant positive associations of moderate magnitude with ankle ROM, with total contraction time exhibiting a similar pattern but of large magnitude. In contrast, eccentric and concentric peak force were inversely associated with ankle ROM, while eccentric power also showed a negative association of moderate magnitude. All remaining relationships, including between-ankle ASYM measures, were not significant and were trivial to small in magnitude (Table 6).

**Table 6:** Relationship between countermovement vertical jump force-time metrics within eccentric and concentric phases of the jumping motion and ankle mobility and between-limb asymmetries in liberos.

Variable [unit]	ROM	ASYM
ECC duration [s]	**0.390** **[0.041]**	0.211 [0.346]
ECC peak velocity [m/s]	0.143 [0.526]	0.203 [0.366]
ECC peak force [N]	**−0.640** **[0.001]**	−0.214 [0.339]
ECC peak power [W]	**−0.416** **[0.046]**	−0.076 [0.736]
CON duration [s]	**0.469** **[0.033]**	−0.076 [0.736]
CON peak velocity [m/s]	0.289 [0.191]	−0.090 [0.690]
CON peak force [N]	**−0.697** **[0.001]**	−0.216 [0.334]
CON peak power [W]	−0.197 [0.380]	−0.160 [0.476]
Contraction time [s]	**0.544** **[0.029]**	0.165 [0.462]
Jump height [cm]	0.293 [0.184]	−0.062 [0.784]
RSI-modified [ratio]	−0.268 [0.228]	0.048 [0.832]
CMJ depth [cm]	−0.288 [0.193]	0.090 [0.692]

Bolded values represent statistically significant relationships (*p* < 0.05).

CM, countermovement; ECC, eccentric; CON, concentric; ROM, range of motion; ASYM, asymmetry.

When examining the same relationships between ankle ROM, ASYM, and force-time metrics during both the eccentric and concentric phases of the jumping motion, including outcome variables such as jump height, RSI-modified, and countermovement depth, no statistically significant associations were observed for outside hitters (Table 7) or middle blockers (Table 8). Specifically, all of the aforementioned relationships were trivial to small in magnitude.

**Table 7:** Relationship between countermovement vertical jump force-time metrics within eccentric and concentric phases of the jumping motion and ankle mobility and between-limb asymmetries in outside hitters.

Variable [unit]	ROM	ASYM
ECC duration [s]	−0.279 [0.094]	−0.309 [0.064]
ECC peak velocity [m/s]	−0.324 [0.051]	−0.321 [0.053]
ECC peak force [N]	0.119 [0.484]	0.222 [0.186]
ECC peak power [W]	0.326 [0.052]	0.223 [0.184]
CON duration [s]	−0.106 [0.531]	−0.253 [0.131]
CON peak velocity [m/s]	0.219 [0.193]	0.084 [0.620]
CON peak force [N]	−0.096 [0.573]	0.098 [0.564]
CON peak power [W]	0.045 [0.789]	0.031 [0.857]
Contraction time [s]	−0.242 [0.149]	−0.311 [0.061]
Jump height [cm]	0.202 [0.231]	0.083 [0.625]
RSI-modified [ratio]	0.319 [0.056]	0.173 [0.306]
CMJ depth [cm]	−0.025 [0.885]	−0.076 [0.660]

CM, countermovement; ECC, eccentric; CON, concentric; ROM, range of motion; ASYM, asymmetry.

**Table 8:** Relationship between countermovement vertical jump force-time metrics within eccentric and concentric phases of the jumping motion and ankle mobility and between-limb asymmetries in middle blockers.

Variable [unit]	ROM	ASYM
ECC duration [s]	0.171 [0.372]	−0.185 [0.336]
ECC peak velocity [m/s]	0.279 [0.142]	0.014 [0.945]
ECC peak force [N]	−0.195 [0.311]	−0.137 [0.480]
ECC peak power [W]	−0.250 [0.192]	−0.112 [0.562]
CON duration [s]	0.014 [0.941]	0.107 [0.581]
CON peak velocity [m/s]	0.015 [0.940]	−0.207 [0.281]
CON peak force [N]	−0.102 [0.598]	−0.101 [0.589]
CON peak power [W]	0.108 [0.577]	−0.117 [0.359]
Contraction time [s]	0.108 [0.579]	−0.071 [0.713]
Jump height [cm]	0.020 [0.919]	−0.182 [0.344]
RSI-modified [ratio]	−0.116 [0.550]	−0.019 [0.920]
CMJ depth [cm]	0.227 [0.236]	0.010 [0.959]

CM, countermovement; ECC, eccentric; CON, concentric; ROM, range of motion; ASYM, asymmetry.

## 4 Discussion

The purpose of the present investigation was to examine the relationship between ankle joint mobility and lower-body force and power-producing capabilities through CMJ across five different playing positions in professional female volleyball players. The main findings indicate that, although ankle ROM was similar across positions, CMJ force-time characteristics varied by playing role, suggesting that ankle mobility may be utilized differently depending on positional demands. When data were pooled across all players, greater ankle ROM was generally associated with lower eccentric and concentric force-producing capabilities. However, these relationships became more nuanced when analyzed by playing position. Specifically, setters and opposite hitters demonstrated inverse associations between ankle ROM and eccentric peak force, concentric peak force and velocity, and jump height, suggesting that greater joint mobility may not necessarily provide a mechanical advantage for maximal force production in these roles. In contrast, liberos exhibited a distinct pattern, whereby greater ankle ROM was directly associated with longer phase durations and total contraction time, but inversely related to eccentric peak force and power, and concentric peak force, reflecting the unique movement demands of their position. Additionally, between-ankle ASYM measures showed no meaningful associations with any CMJ performance metrics across positions, indicating a limited role of ankle mobility ASYM in vertical jump mechanics within this cohort of athletes. Overall, these findings demonstrate varying patterns of association between ankle mobility and CMJ performance across playing positions, which may reflect differences in position-related movement demands and strategies.

Setters constitute a subgroup of players whose primary responsibility is to coordinate the offensive strategies of the game (24, 25, 36). Although setters perform the greatest number of jumps during matches, these jumps are typically of low intensity and tend to be symmetrical (10, 37, 38). Rather than emphasizing maximal jump height or force production, setters’ jumps are primarily executed to facilitate precise ball placement and optimal timing with attackers (i.e., synchronization with the approach and jump of the attacker), often from constrained or non-ideal body positions. Therefore, neuromuscular demands for setters may prioritize movement control, joint stability, and repeatability over maximal force and power production (39, 40). In this context, the observed inverse associations between ankle ROM and eccentric and concentric force outputs, along with jump height, imply that increased ankle mobility can support more controlled movement execution rather than explosive force generation for this volleyball-specific position.

The libero is a specialized position in volleyball, primarily responsible for serve reception and defensive actions, which require frequent low stances and rapid adjustments of body alignment (39, 41). As such, players in this position rely heavily on postural stability, balance control, and agility rather than maximal vertical jump performance (39). Adequate ankle ROM is therefore essential to facilitate stable low stances and effective force modulation during defensive actions (39). In the present study, liberos primarily exhibited positive associations between ankle ROM and both eccentric and concentric phase durations during the CMJ, indicating that greater ankle mobility was associated with longer movement execution times and a deeper and more controlled countermovement strategy. In addition, negative correlations between ankle ROM and eccentric and concentric peak force indicate that increased countermovement depth may be associated with lower force magnitudes, reflecting a movement strategy that prioritizes controlled force absorption over rapid force production. This interpretation is in direct agreement with the findings of Giardullo et al. (42), who reported that greater ankle mobility is accompanied by reduced explosive strength and lower rates of force development, reflecting neuromuscular strategies that favor controlled loading over maximal force output. Also, given that liberos are generally characterized by shorter stature (39, 41, 43), anthropometric factors such as limb length and body mass may partially contribute to these relationships, although these variables were not directly examined in the present study. Importantly, these findings may reflect adaptations associated with the functional demands of the libero role, where controlled movement strategies and stability are prioritized over maximal explosive output.

The opposite hitter is a critical playing position in volleyball, primarily recognized for its demanding attacking role and frequent involvement in high-intensity offensive and blocking actions (44, 45). Players in this position regularly execute maximal and highly explosive jumps, including back-row attacks, which require substantial force and velocity generation within limited time frames (10, 44–46). Given these performance demands, the present investigation found that greater ankle ROM was inversely related to concentric peak velocity, force, and power, as well as RSI-modified and jump height in opposite hitters. This suggests that excessive ankle mobility may be mechanically disadvantageous for explosive jump performance, as it could reduce the efficiency of force transmission and elastic energy reutilization during the stretch-shortening cycle, ultimately impairing force and power-producing capabilities (42, 47). Given the reactive and explosive demands of the opposite hitter role, elevated levels of ankle mobility may not be necessary to support optimal jump performance. Moreover, opposite hitters are typically among the tallest and heaviest players on the court, and such anthropometric characteristics may further influence jumping strategy by favoring knee and hip-dominant movement patterns over ankle-driven propulsion (48–50). Therefore, these results highlight the need to examine the contribution of proximal joints (e.g., knee and hip) and overall movement strategy, in addition to ankle mobility, when assessing jumping mechanics in this position.

Outside hitters represent one of the most variable roles in volleyball, combining responsibilities in serve reception, front and back-row attacking, and transitional play, which requires continuous adaptation of movement strategies rather than reliance on a uniform motor pattern (7, 51). Accordingly, the absence of meaningful relationships between ankle ROM and CMJ force-time variables in outside hitters likely reflects the heterogeneous demands of this position, where performance is determined by the ability to efficiently adjust movement strategies across diverse competitive scenarios rather than by a single biomechanical factor. Furthermore, middle blockers constitute a highly specialized front-row role and execute a high volume of jumps at near-maximal intensity during attacking and blocking actions, often under strict temporal constraints (7, 51). For this position, timing, reaction speed, and body height may represent more influential determinants of performance than isolated measures of ankle mobility. The lack of significant associations observed in middle blockers may therefore reflect the limited ecological validity of bilateral CMJ assessments, as game-specific jumping frequently involves lateral displacement, unilateral take-offs, and rapid decision-making. While further research is warranted on this topic, these findings suggest that the absence of position-specific relationships among outside hitters and middle blockers may likely be attributed to the complex and task-specific nature of volleyball-specific demands rather than to limitations in physical capacity (e.g., ankle mobility).

Moreover, no significant associations were observed between inter-limb ankle ASYM and CMJ force-time characteristics, irrespective of playing position. Although isolated small correlations were identified, none reached the level of statistical significance, indicating a limited association between ankle asymmetry and CMJ performance in professional female volleyball players. These findings suggest that, within a well-trained cohort, variations in ankle ASYM may not constitute a primary constraint on CMJ performance, as athletes likely compensate through coordinated movement strategies and greater contributions from proximal joints (52–54). In addition, it should be noted that a certain degree of inter-limb ASYM appears to be a common characteristic in team sports and does not inherently reflect impaired neuromuscular function or performance capacity (52, 55). In particular, moderate ASYM magnitudes (5%–15%) are often considered sport-specific adaptations resulting from the specialized, repetitive, and frequently unilateral demands of training and competition (52, 54–56). When asymmetry remains within these ranges, it should not be immediately linked to decrements in athletic performance. Collectively, these findings indicate that ankle mobility ASYM, when considered in isolation, may have limited functional relevance for CMJ performance in well-trained professional volleyball players.

Lastly, it should be noted that when compared to previous studies reporting significant associations between ankle dorsiflexion and jump performance (17, 19), the current cohort demonstrated relatively high ankle ROM values, which are indicative of well-developed mobility profiles. For instance, Patti et al. (19) reported mean ankle dorsiflexion values of approximately 29–31° in amateur female volleyball players, whereas the professional athletes examined in the present investigation exhibited substantially greater values, ranging from 40 to 43°. These discrepancies may be attributed to methodological differences, including the technology used to assess ankle ROM (i.e., manual vs. digital goniometer) and the body positions adopted during dorsiflexion testing (e.g., seated or half-kneeling), as well as participant-related factors such as age (i.e., 16 vs. 21 years) and level of play (i.e., amateur vs. elite). Taken together, these findings suggest that once a sufficient level of ankle mobility is achieved, further increases may have limited relevance for jumping performance, particularly in well-trained professional players.

While offering a deeper insight into lower-body biomechanical characteristics of professional female volleyball players, this study is not without limitations. Ankle ROM was assessed under controlled conditions that may not fully reflect joint behavior during sport-specific jumping tasks involving approach steps, unilateral take-offs, or lateral movements. Likewise, the bilateral CMJ represents a standardized laboratory assessment and may not capture the mechanical and coordinative demands of volleyball-specific actions such as spike approach jumps. The use of this task may therefore have masked functional inter-limb asymmetries due to compensatory strategies. In addition, joint mobility at the knee and hip was not directly assessed, despite the potential importance of proximal joint contribution for jump performance in certain playing positions. Given the number of statistical comparisons performed without adjustment for multiple testing, an increased risk of Type I error cannot be excluded. Therefore, the position-specific findings should be considered exploratory and interpreted as potential trends or associations that warrant confirmation in larger samples rather than as definitive effects. Additionally, anthropometric characteristics were not included as covariates and may have contributed to the observed associations, particularly given the differences in body size across playing positions. Thus, future research should incorporate sport-specific jumping tasks and multi-joint assessments to better reflect the positional and movement-specific demands of volleyball performance.

## 5 Practical application

Although ankle ROM was generally comparable across playing positions, its relationship with CMJ force-time characteristics differed according to positional demands in professional female volleyball players. Greater ankle mobility was associated with lower eccentric and concentric force-producing capabilities when all athletes were analyzed collectively; however, the patterns of association varied across playing positions. Setters and opposite hitters demonstrated inverse associations between ankle ROM and force, velocity, power, and jump height, suggesting that excessive ankle mobility may not be advantageous for explosive jump performance in positions requiring rapid force transmission and high-intensity jumping actions. In contrast, liberos exhibited longer eccentric and concentric phase durations and reduced force outputs with greater ankle mobility, indicating movement strategies that prioritize control and stability over maximal force production. No meaningful relationships were observed in outside hitters or middle blockers, suggesting that ankle mobility may play a less prominent role in CMJ performance for these positions. From a practical perspective, coaches and practitioners should avoid applying generalized ankle mobility recommendations across all volleyball athletes and instead interpret ROM characteristics within the context of position-specific neuromuscular demands. Additionally, inter-limb ankle ASYM did not appear to meaningfully influence bilateral CMJ performance in this well-trained cohort, indicating that small ASYM in ankle mobility may not require corrective intervention in the absence of performance limitations or injury history.

## Data Availability

The datasets presented in this article are not readily available because due to restrictions regarding participant confidentiality and the protection of sensitive, personally identifiable information. Requests to access the datasets should be directed to dcabarkapa@ku.edu.
